# A statistical approach to white-nose syndrome surveillance monitoring using acoustic data

**DOI:** 10.1371/journal.pone.0241052

**Published:** 2020-10-22

**Authors:** Lorin L. Hicks, Nathan A. Schwab, Jessica A. Homyack, Jay E. Jones, Bryce A. Maxell, Braden O. Burkholder

**Affiliations:** 1 Weyerhaeuser, Kalispell, MT, United States of America; 2 Tetra Tech, Missoula, MT, United States of America; 3 Weyerhaeuser, Centralia, WA, United States of America; 4 Weyerhaeuser, Seattle, WA, United States of America; 5 Montana Natural Heritage Program, Helena, Montana, United States of America; Bowling Green State University, UNITED STATES

## Abstract

Traditional pathogen surveillance methods for white-nose syndrome (WNS), the most serious threat to hibernating North American bats, focus on fungal presence where large congregations of hibernating bats occur. However, in the western USA, WNS-susceptible bat species rarely assemble in large numbers and known winter roosts are uncommon features. WNS increases arousal frequency and activity of infected bats during hibernation. Our objective was to explore the effectiveness of acoustic monitoring as a surveillance tool for WNS. We propose a non-invasive approach to model pre-WNS baseline activity rates for comparison with future acoustic data after WNS is suspected to occur. We investigated relationships among bat activity, ambient temperatures, and season prior to presence of WNS across forested sites of Montana, USA where WNS was not known to occur. We used acoustic monitors to collect bat activity and ambient temperature data year-round on 41 sites, 2011–2019. We detected a diverse bat community across managed (n = 4) and unmanaged (n = 37) forest sites and recorded over 5.37 million passes from bats, including 13 identified species. Bats were active year-round, but positive associations between average of the nightly temperatures by month and bat activity were strongest in spring and fall. From these data, we developed site-specific prediction models for bat activity to account for seasonal and annual temperature variation prior to known occurrence of WNS. These prediction models can be used to monitor changes in bat activity that may signal potential presence of WNS, such as greater than expected activity in winter, or less than expected activity during summer. We propose this model-based method for future monitoring efforts that could be used to trigger targeted sampling of individual bats or hibernacula for WNS, in areas where traditional disease surveillance approaches are logistically difficult to implement or because of human-wildlife transmission concerns from COVID-19.

## Introduction

North American bat species face several contemporary conservation challenges throughout their range, including threats to habitat from loss and alteration of forested environments, and direct mortality from development of wind energy infrastructure [1, 2]. However, transmission of *Pseudogymnoascus destructans* (Pd), the pathogen that causes white-nose syndrome (WNS), has emerged as the most serious threat to over a dozen species of North American bats that use caves and mines for hibernacula [3]. The Pd fungus invades skin tissues (e.g., wing membrane) which disrupts homeostasis and causes dehydration that corresponds to an abnormally high frequency of arousals from torpor [4]. Bats with WNS increase arousal [5] and acoustic activity rates [6] above baseline levels during the normal hibernation period, which depletes critical energy reserves and can ultimately cause mortality. WNS has caused precipitous declines in populations of hibernating bats and has contributed to the federal listing of the northern long-eared bat (*Myotis septentrionalis)* as threatened under the U.S. Endangered Species Act [7] and endangered listings for three bat species in Canada under the Species at Risk Act [northern long-eared bat, little brown bat (*Myotis lucifugus*), and tri-colored bat (*Perimyotis subflavus*)] [8]. Monitoring for WNS is a critical management action and primarily has focused on detecting the Pd pathogen either directly from bats captured in hibernacula or with environmental samples from occupied caves and mines [9].

Since its emergence in eastern North America in 2006, WNS has spread westward with some models predicting WNS to arrive to Montana by 2026 [10]. In June 2020, sampling conducted at bridges in 3 eastern Montana counties confirmed the presence of Pd, but not WNS [11]. Further, WNS was detected in a little brown bat in Washington state in 2016 and Pd detection continues to increase among sites and across different species within Washington [12]. Thus, there is a second WNS epicenter, which could affect known and previously unrecognized susceptible bat species and increase the spread of WNS through western North America from both the west and the east [13].

The presence of WNS in the Pacific Northwest opens another front in critical efforts to combat bat declines and shortens the timeline for implementing conservation strategies to protect vulnerable bat species. This effort is complicated by the fact that winter hibernation behavior for most western species is poorly understood [14], yet this is when WNS has the greatest negative impacts on bat populations [2, 15, 16]. Further, little is known about winter roost sites and activity patterns of many bats in western North America. A recent review indicates that WNS-susceptible species rarely congregate in large numbers in the western U.S. and that known winter roosts may be relatively uncommon or difficult to identify [17]. Thus, typical surveillance approaches of estimating hibernating bat numbers and sampling for Pd implemented in eastern North America where large hibernacula are more common does not translate well to all ecoregions [18]. Therefore, alternate approaches to facilitate pathogen surveillance, monitor disease impacts, and conduct mitigation efforts for WNS are urgently needed [13].

In addition to WNS, bats can be influenced by habitat alteration from anthropogenic activities, such as forest harvesting [19]. Forested areas provide essential habitat features that support bat diversity, including roost sites (e.g., trees) and foraging areas (e.g., riparian zones, wetlands) [20–22]. Sustainable management of these forest resources may minimize the environmental stress on bats outside of the winter season (when WNS most severely impacts bats). Despite a robust network of public lands managed for preservation in western North America [23], bat species richness is low in some protected areas, such as Glacier National Park [24] and lower bat activity is commonly reported in undisturbed habitats compared to disturbed [19]. Managed forests support many species of bats not found or poorly represented in protected areas on public lands [1] and some forest management practices that reduce understory vegetation such as commercial thinning and prescribed burning can increase bat activity and occupancy [19, 25, 26]. Nationally, public and private forestlands are managed under state forest practices rules, water quality best management practices, and forest sustainability programs (i.e., Sustainable Forestry Initiative, Forest Stewardship Council, American Tree Farm) to protect water quality and quantity, which includes riparian buffers that often provide habitat for bats.

We quantified presence of bats across forested sites in Montana, USA to document baseline levels of species diversity and activity rates prior to presence of WNS. Our objective was to evaluate long-term acoustic monitoring as a surveillance approach to monitor for potential WNS presence in overwintering bat populations on both managed and unmanaged forest. Specifically, we developed a statistical method to evaluate whether activity data occur outside the range of normal conditions, thus warranting more intensive sampling for the presence of Pd on bats or in the environment. We predicted that bat populations in the western U.S. would have low levels of activity during winter months, high levels of activity during the summer, and that acoustic monitoring could be used as an effective approach for disease surveillance in remote areas of the Intermountain West.

## Methods

### Study area

**Fig 1:**
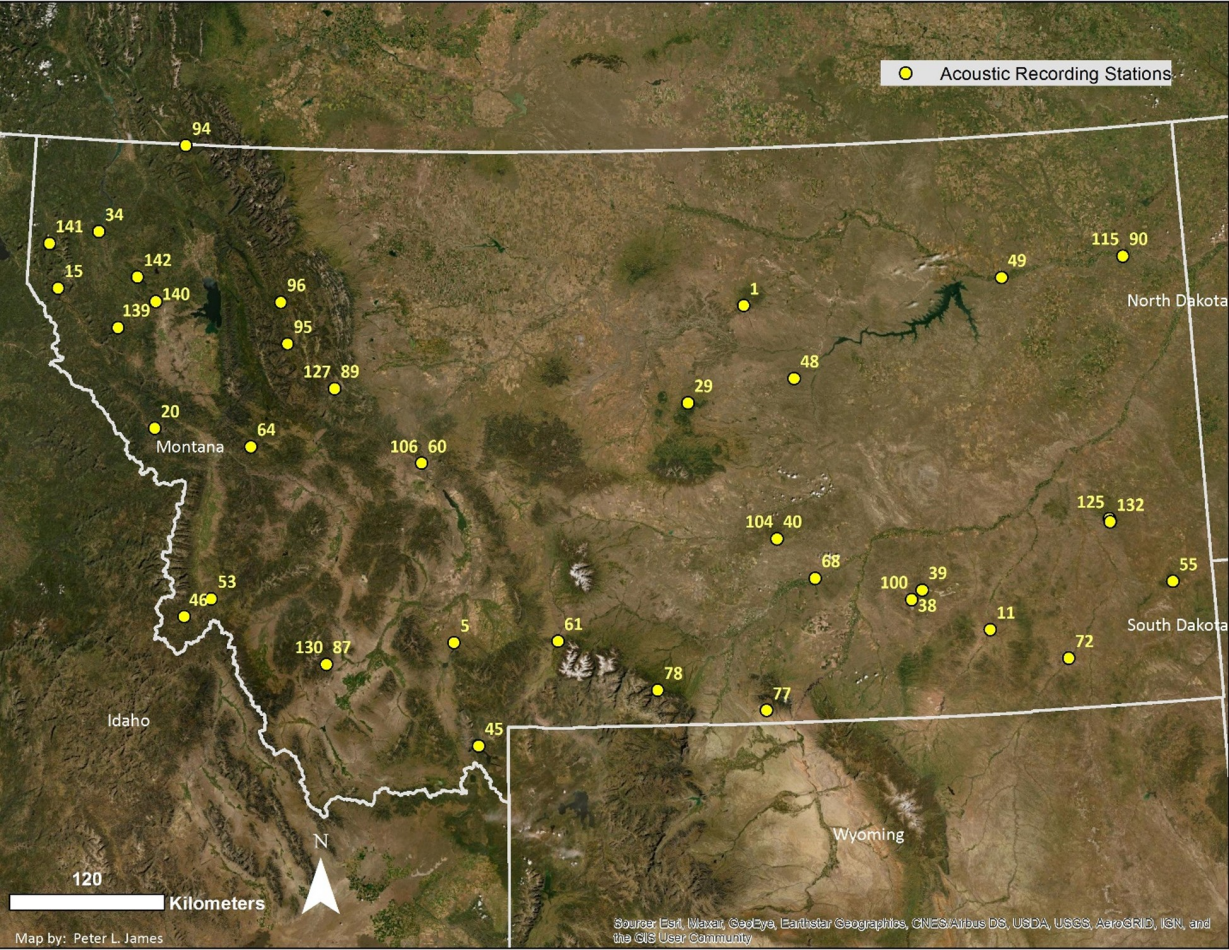
We passively monitored bat activity with acoustic recording devices across 41 sampling sites in Montana, USA, from 2011–2019. Reprinted from ESRI with permission from ESRI, under a CC BY 4.0 license, original copyright 2020.

Our study area included forested areas across Montana, USA (Fig 1). The forested areas in the western portion of the state were Douglas fir (P*seudotsuga menziesii*)/snowberry (*Symphoricarpos albus*) forest type, which generally characterized the region [27] and included locations of our acoustic detector stations. These forests had a mixed overstory, including Douglas-fir, ponderosa pine (*Pinus ponderosa*), grand fir (*Abies grandis*), western larch (*Larix occidentalis*), lodgepole pine (*Pinus contorta*), and Engelmann spruce (*Picea engelmannii*), with understory vegetation comprised of snowberry, huckleberry (*Vaccinium spp*.) and beargrass (*Xerophylum tenax*). Westside elevations ranged from 800 to 1150 meters. The climate in these ecoregions was continental-maritime with relatively low annual precipitation, with most precipitation occurring as snow [28]. The forested locations in eastern Montana were characterized by limber pine (*Pinus flexilus*) and ponderosa pine (*Pinus ponderosa*) forest with understory of Idaho fescue (*Festuca idahoensis*) and bluebunch wheatgrass (*Agropyron spicatum*). Eastside sample site elevations ranged from 850–1520 meters. Climate in the eastern ecoregions was continental with dramatic fluctuations in winter temperatures and longer, hotter, more arid summers than western ecoregions [29].

We selected 41 sampling sites that were near lentic or lotic systems and had ≥25% forest cover within a 1-kilometer buffer, estimated from the Montana Land Cover data set in a geographic information system [30] (Fig 1). We did not randomly select sites for acoustic detectors but installed them in areas with potential for high bat activity that included proximity to standing live and dead trees, talus and rock cliffs, and waterbodies. We located four sites on intensively managed forest owned and managed by Plum Creek Timber Company (after 2016, Weyerhaeuser) for production of sawtimber and other forest products. The remaining 37 sampling sites were selected from a regional bat acoustic monitoring network organized by the Montana Natural Heritage Program [31, 32]. These locations are primarily represented by federal and state landownership, but also include some tribal and private land. We received permissions from the relevant regulatory body or private landowners to conduct the research on all study sites, including the Northern Region of the U.S. Forest Service, located in Missoula, Montana USA; Montana/Dakotas State Bureau of Land Management, located in Billings, Montana USA; Montana Department of Fish, Wildlife, and Parks, located in Missoula, Montana USA, and numerous private landowners.

### Field sampling

We installed a single, full-spectrum acoustic recording unit (ARU) at each sampling site. Not all ARUs recorded data simultaneously or across the entire 2011–2019 study period, but we installed units for year-round recording of bat echolocation. Each ARU station consisted of a SM3BAT or SM2BAT+ acoustic detector (Wildlife Acoustics, Maynard, Massachusetts) and a microphone (U1 or SMX-US) mounted on a pole at a height of approximately 3 meters. The ARUs operated on a 6- or 12-volt charging system (20-watt solar panel, 36-amp hour battery, and a charge controller) and housed in a weather-proof container on the ground below the microphone. We set the ARUs to record from sunset to sunrise to maximize battery life and balance with other logistical restraints of year-round sampling in remote locations. The units employed a sampling rate (samples per second) of 196,000, 256,000 or 384,000 kHz. All ARUs used a minimum frequency filter of 8 kHz, a trigger window of 2 seconds and a maximum file length of 5–8 seconds. Most ARUs recorded in the WAC0 compression format while four ARUs recorded directly to.wav format. The individual ARU equipment and settings varied between the hardware platforms (SM3BAT vs. SM2BAT+), with detection distances of approximately 20–30 meters [33], depending on the frequency of the source and other environmental variables (i.e., temperature and humidity) [34]. The equipment and settings were consistent for a given site throughout the study. The sensitivities of all microphones were within the manufacturer’s specifications at the beginning of the deployment at each sampling site and replaced approximately annually. We examined equipment for functionality, checked microphone sensitivity, and exchanged data storage cards approximately once per month during summer and up to 6 months between visits during winter due to logistical constraints of access. Each ARU recorded a site-specific temperature approximately once every 1 or 5 minutes.

### Data analysis

At the end of our study, we parameterized acoustic files with Sonobat 4.1.0 (Sonobat, Arcata, California USA) using a high-performance computing cluster at Montana Technological University. The parameters were then input into the Sonobat Montana [20160912] classifier to automatically assign a species classification to each bat pass. The Montana classifier uses two regional species suites, each tuned to the species potentially present in that portion of the state. In cases where a bat pass was unable to be classified to species, a generic “bat” label was assigned. Similar to the North American Bat Monitoring Program [35], we defined a bat pass as a sequence of echolocation pulses separated by more than 2 seconds of silence. We limited the maximum file duration to 5–8 seconds to reduce potential for recording more than one bat per sequence [36]. To quantify activity rates of bats, we analyzed the number of bat passes per detector-night (the number of operational detectors multiplied by the number of nights). For a night to be included in our estimates of detector- nights, the detector needed to register an internal temperature in the status file. We excluded data that did not meet this criterion from analysis. We manually verified a subset of diagnostic bat passes (approximately monthly for each species) with Sonobat to ensure that species diversity of bats was accurate at all sampling stations [37].

We calculated the average number of passes per night over each month, by species, site and year using the ratio of total monthly qualified passes and the number of nights in the month. Similarly, we calculated the average number of passes per night across all species, including passes without a species classification (total bat activity). A mean monthly temperature (°C) was estimated from the mean of temperatures recorded at each site between sunset and sunrise across all nights in a month.

We used a statistical model to describe variation in monthly-average nightly bat activity associated with several sources. To address our research questions, we aimed to characterize the extent to which activity varied among sampling sites, across years, by season, and in association with temperature. We fit a linear mixed-effects model with fixed effects for monthly-average mean temperature, season, and their interaction. We included random intercepts by site and year and random slopes for the temperature effect by site and year. Thus, this model allowed for different overall levels of bat activity by site and year, and different temperature trends by site and year, while estimating population-average effects and quantifying the variation among sites and years. Bat activity was log-transformed prior to analysis, and a constant of 0.01 was added prior to transformation to accommodate values of zero. We defined season by assigning each month to one of four calendar-based seasons (Winter = Dec, Jan, Feb; Spring = Mar, Apr, May; Summer = Jun, Jul, Aug; Fall = Sep, Oct, Nov). We fit all models in R [38] using package lme4 [39]. We excluded sites with < ten months with at least one bat detection across all species from data used to fit the model. Additionally, only data from 2012–2016 were used to fit the model, as limited data were available outside of this range. We note that both data restriction choices were performed to ensure sufficient data were available to estimate site-specific effects in the mixed effects model, but that no such restrictions were used in our raw data summaries. In addition to using the fitted model to describe variation in bat activity, we also used the model to generate prediction intervals [40] for new or future observations of bat activity in our region. We generated prediction intervals based on the model output using the ‘predictInterval’ function in package merTools [41].

## Results

We collected acoustic data used for our analysis between 28 Sept 2011 and 4 August 2019, totaling 24,850 detector-nights at 41 sites. Stations operated for an average of 606 (SD = 307) nights through the study. There were gaps in recordings because of equipment malfunction and wildlife encounters, but more than 5.37 million bat passes were recorded. After removing months with erroneous data due to equipment malfunction, 868 months of bat pass and temperature data were analyzed (per site mean: 21.2 months, SD = 10.7 months). We manually vetted acoustic files to confirm 13 species across the study, 11 of which were observed on managed forest sites (Table 1). Three species [big brown bat (*Eptesicus fuscus*), silver-haired bat (*Lasionycteris noctivagans*), and western small-footed myotis (*Myotis ciliolabrum*)] had confirmed activity in all calendar months, indicating that some bat species maintained some level of flight activity year-round. Three presumed migratory species–spotted bat (*Euderma maculatum*), eastern red bat (*Lasiurus borealis*), and hoary bat (*L*. *cinereus*)–were observed in 8, 6, and 7 months, respectively. Of the 5.37 million bat detections, approximately 32% were auto-classified to species by the Sonobat Montana [20160912] classifier, which is consistent with the level of species identification from other research [2]. Due to the high percentage of missing species information, our analyses include both estimates of total bat activity, i.e., total activity over all species, including bat passes not identified to species, and estimates for individual species where possible.

**Table 1:** Summary of bat activity across forested sites in Montana using acoustic recording units deployed year-round from 2011–2019.

Species	Total filtered auto-identified detections	Sites with manually confirmed presence (n = 41)	Calendar months with manually confirmed presence (n = 12)
*Corynorhinus townsendii*	286^a^	21	5
***Eptesicus fuscus***	419,335	34	12
*Euderma maculatum*	527	8	8
*Lasionycteris noctivagans*	364,152	37	12
*Lasiurus borealis*	104,724^b^	16	5
*Lasiurus cinereus*	262,867	39	7
***Myotis californicusck***^c^	112,147	15	9
***Myotis ciliolabrum***^c^	100,889	32	12
***Myotis evotis***	52,912	39	9
***Myotis lucifugus***	221,886	39	10
***Myotis thysanodes***	3,750	16	7
***Myotis volans***	42,001^d^	7^d^	3^d^
***Myotis yumanensis***	44,495	15	8
(unidentified)	3,645,255	NA	NA

Bolded species are known or suspected to be affected by WNS; underlined species are documented carriers of *Pseudogymnoascus destructans* without WNS [42].

^a^ Rarely detected away from roosts due to very quiet calls; auto-identified calls may be confused with other species.

^b^ Auto-identified calls are frequently confused with *M*. *lucifugus*; most of these calls may be *M*. *lucifugus*.

^c^ *M*. *californicus* and *M*. *ciliolabrum* not yet been documented with WNS, but are both believed to be susceptible given impacts to closely related *M*. *leibii* in the eastern U.S.

^d^ Definitive characteristics for hand confirmation rarely encountered for *M*. *volans*; unable to assess accuracy of auto-identified call sequences, but proportion of call volume appears consistent with proportion of capture data in Montana.

Across 24,850 detector-nights of sampling ambient temperatures, mean monthly temperatures ranged from –16.5°C to 23.3°C. Mean monthly temperatures (± SD°C) in Montana varied across seasons [Winter -1.7°C (± 3.9°C); Spring 5.8°C (± 4.3°C); Summer 16.2°C (± 3.2°C); and Fall 6.7°C (± 5.7°C]).

**Fig 2:**
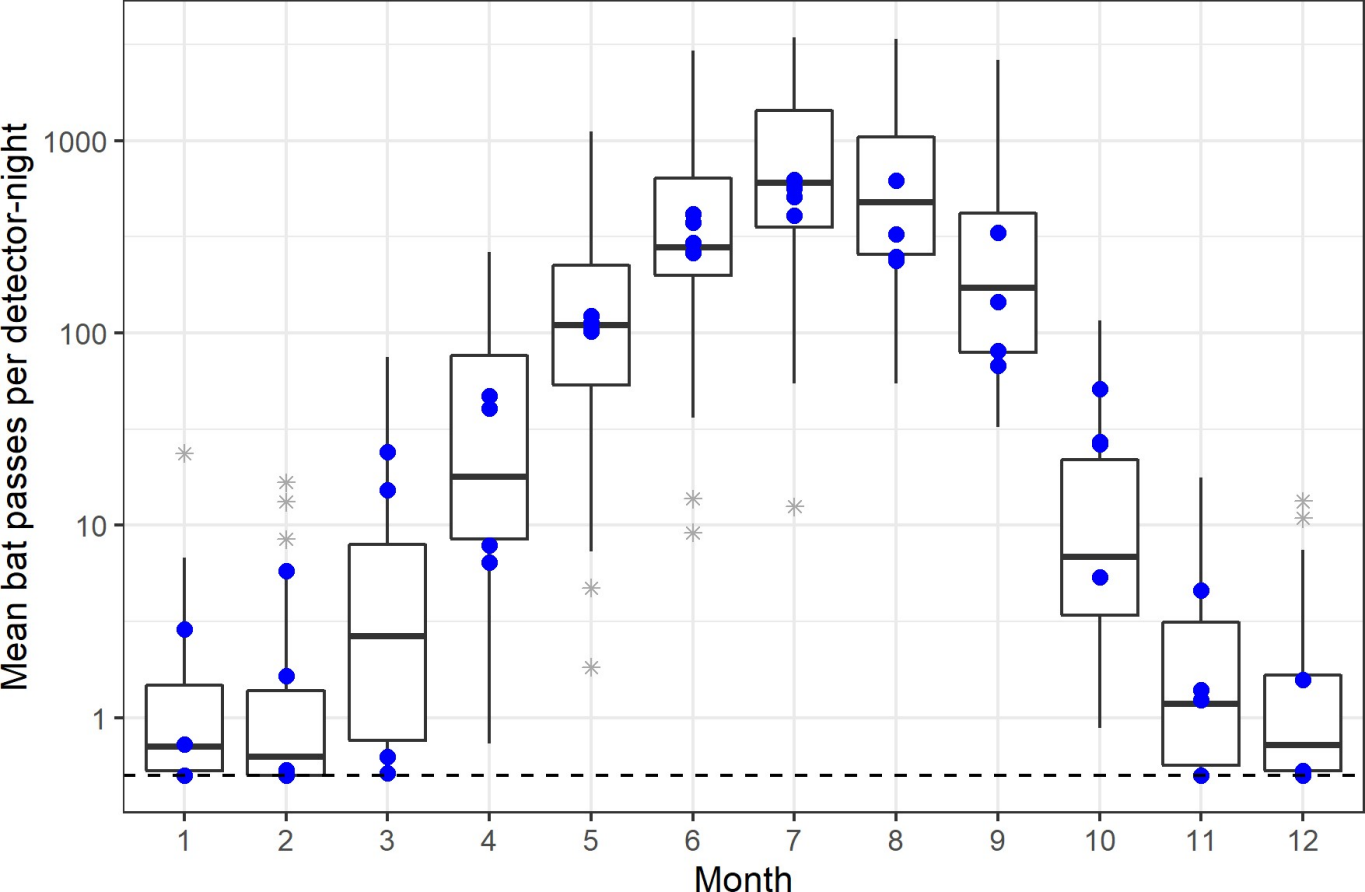
Among-site distribution of mean per-night bat activity by month, displayed on the log-10 scale. Bat activity is summed across all species and averaged over all years for 41 unique sites and 437 site-months. Boxplots display mean, quartile, and max and min values of site averages, with asterisks indicating possible outliers. Month number follows the calendar months with January represented by 1. Solid blue points represent values for managed stands. An offset of 0.5 was added to all points to aid with visual display of 0 values on the log scale. The horizontal dashed line indicates values of zero.

**Fig 3:**
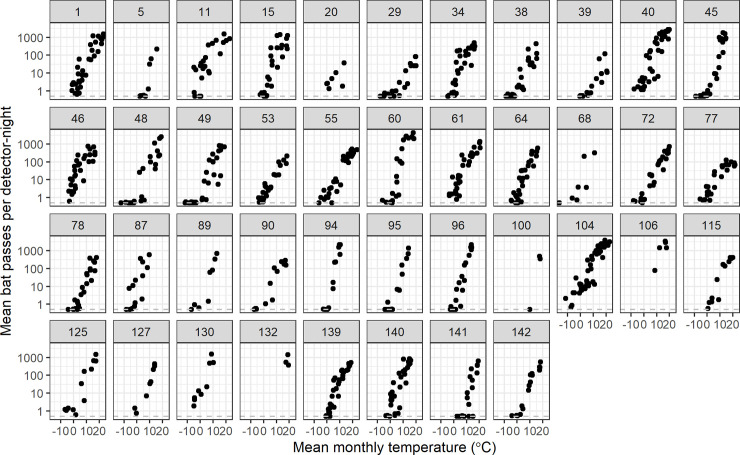
Monthly average bat passes per day vs. mean monthly temperature (n = 868 site months). Data for each site are shown in separate panels, labelled with the site ID number. An offset of 0.5 was added to all points for plotting on the log-10 scale. The horizontal dashed line indicates values of zero. Each black point represents a unique year-month combination.

Bat activity tended to be highest during summer months with most sites averaging >100 passes/night during July and August (Fig 2). Additionally, most sites showed some activity during December and January, with one site averaging >10 passes/night. Species-specific results show similar trends. We observed a strong positive association between average total bat activity and mean monthly temperature (Fig 3). The relationship was approximately log-linear for all 41 study sites, although the level of activity varied by site.

**Fig 4:**
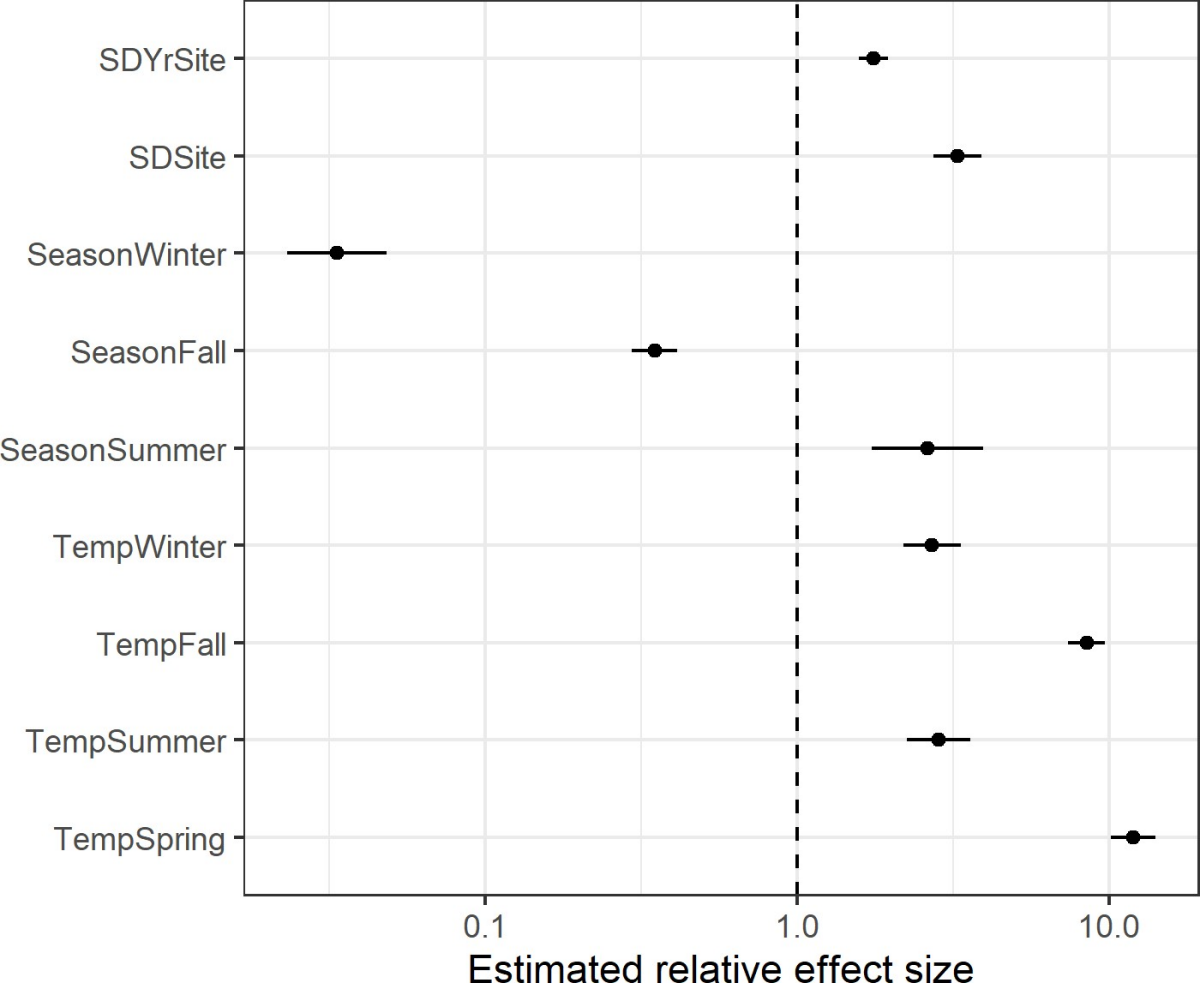
Model estimates of relative effect sizes for selected fixed and random effects. Estimates indicate relative (multiplicative) differences in expected mean total bat activity across all species, such that a value of 10 represents 10x greater activity and 0.1 represents 10x lower activity. Parameters with a ‘Temp’ prefix show the expected difference in response for a 5°C temperature increase by season. Parameters with a ‘Season’ prefix show the expected difference from Spring activity at a constant temperature of 7.3°C. Parameters with a ‘SD’ prefix represent model random effects and show the estimated 1-SD variation in mean response among sites and among years within a site. Horizontal lines show +/- 1 standard error bounds. The vertical dashed line at 1.0 indicates no relative difference in bat activity.

**Fig 5:**
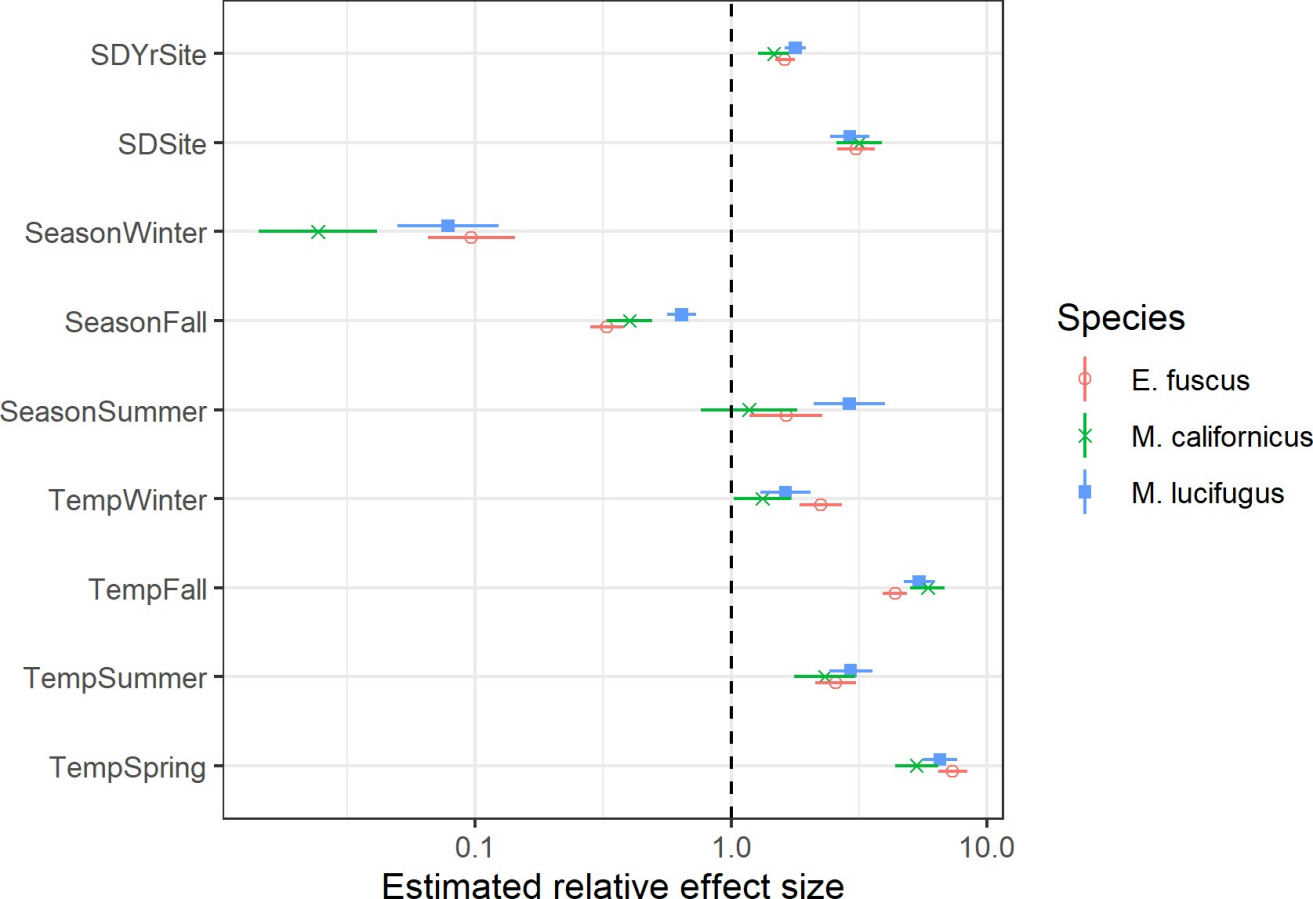
Model estimates of relative effect sizes for selected fixed and random effects from three species-specific models. Results for each species are shown with different colors and symbols. Parameters with a ‘Temp’ prefix show the expected difference in response for a 5°C temperature increase by season. Parameters with a ‘Season’ prefix show the expected difference from Spring activity at a constant temperature of 7.3°C. Parameters with a ‘SD’ prefix represent model random effects and show the estimated 1 SD variation in mean response among sites and among years within a site. Horizontal lines show +/- 1 standard error bounds. The vertical dashed line at 1.0 indicates no relative difference in bat activity.

**Fig 6:**
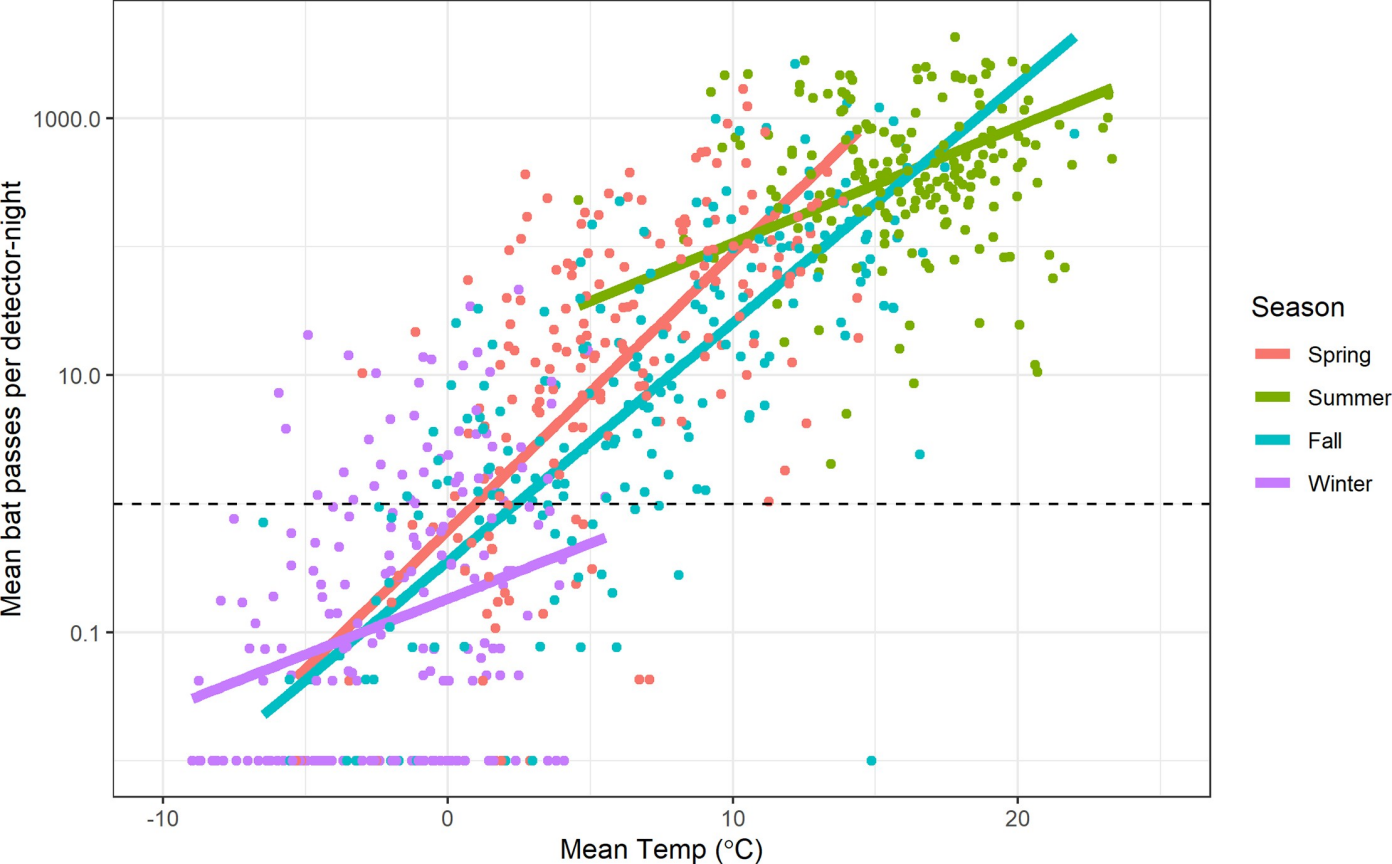
Mean bat passes per detector-night across all species vs. mean monthly temperature. Each point represents a unique site month (n = 868). Points are color coded by calendar season with Winter = Dec, Jan, Feb; Spring = Mar, Apr, May; Summer = Jun, Jul, Aug; Fall = Sep, Oct, Nov. Solid lines show model estimated mean trends with all random effects set to zero. Note that an offset of 0.01 is added to the mean passes per detector-night to allow plotting on the log10-scale.

Results from our fitted model indicated a strong positive association between mean monthly temperature and total bat activity that varied by season (Figs 4 and 5). The estimated trends during the fall and spring indicated approximately 12.0- and 8.5-times greater numbers of bat passes per day, respectively, for every 5°C increase in mean monthly temperature, on average, after adjusting for site and inter-annual differences. The association between bat activity and temperature was less pronounced during the winter and summer, where in both seasons we estimated approximately 2.8-fold greater activity, respectively, for 5°C increases in temperature. Overall levels of activity at a similar temperature also varied by season, although the difference was strongly dependent on temperature (Fig 6).

We used the model random effects to estimate among-site and among-year variation in the mean response and temperature trends. We estimated considerable among-site variation in total bat activity after adjusting for temperature and seasonal effects, with site-specific means varying approximately 3.3-fold (1 SD) and among-year variation within a site of approximately 1.8-fold (Fig 4). Estimated temperature trends among sites and among years were generally consistent, with variation in slopes of approximately 12% and 1% respectively.

We fit this same model form to three individual species–*Eptesicus fuscus*, *Myotis lucifugus*, and *M*. *californicus*–to explore the possibility of seasonal differences in temperature associations among a species less vulnerable to WNS [*E*. *fuscus;* [43]], one highly vulnerable (*M*. *lucifugus;* [44] and one of unknown vulnerability (*M*. *californicus*). We note that since the majority of the bat passes were unidentified, the species-specific estimates may contain substantial unknown biases, as an unknown proportion of the unidentified bat passes consist of these species. Our results suggest that winter activity for *M*. *californicus* is less strongly associated with mean monthly temperature than *E*. *fuscus*, while the reverse is suggested for the fall (Fig 5).

**Fig 7:**
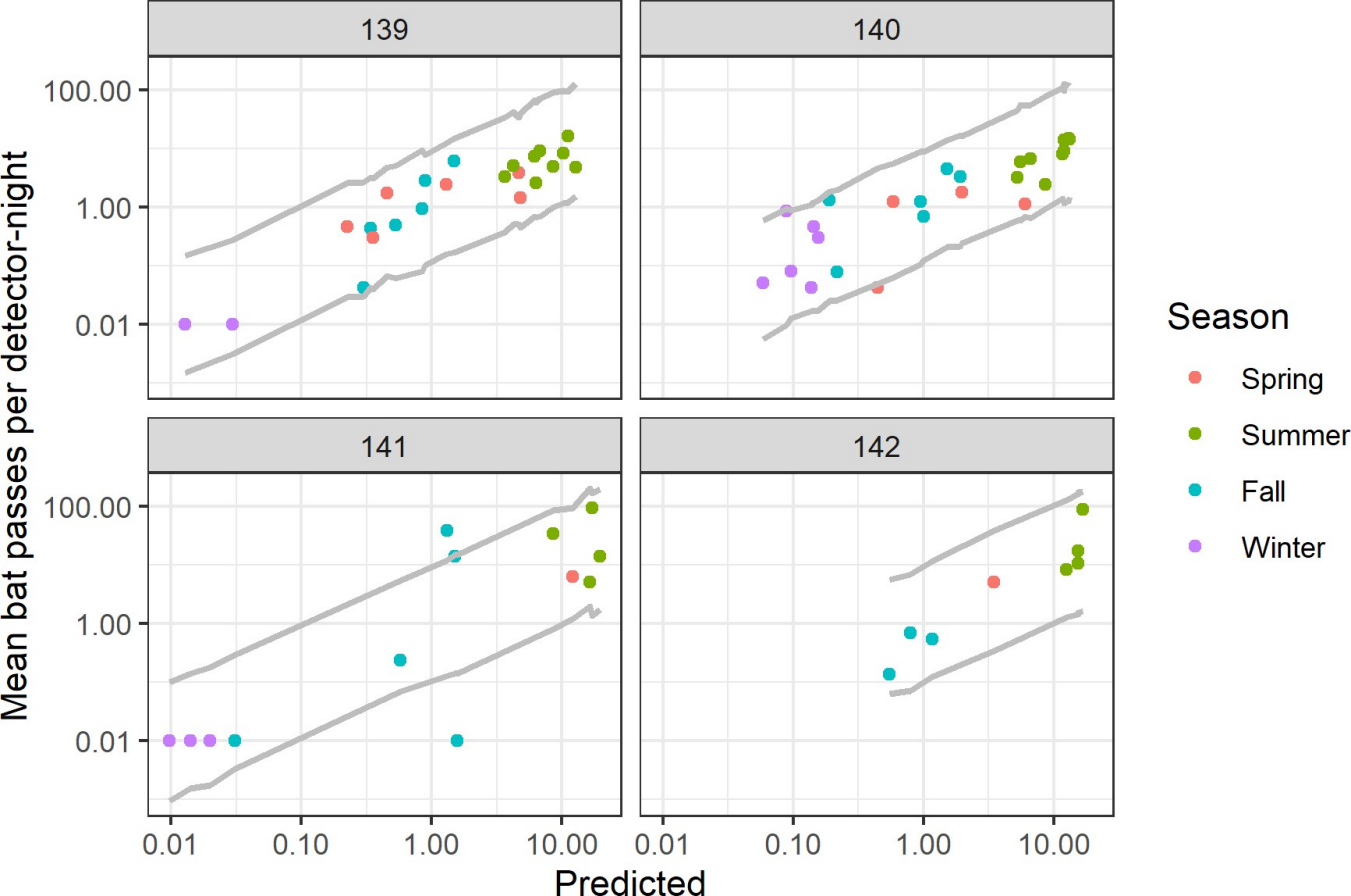
Monthly mean bat passes per detector-night vs. model predictions on four forested sites for the species *E*. *fuscus*. Points are color-coded by calendar season with Winter = Dec, Jan, Feb; Spring = Mar, Apr, May; Summer = Jun, Jul, Aug; Fall = Sep, Oct, Nov. Solid grey lines represent site-specific 95% point-wise prediction intervals. The prediction intervals are not smooth due to differences in uncertainty for data collected in different seasons. Note that an offset of 0.01 is added to the mean passes per detector-night to allow plotting on the log10-scale. Additional data collected for these sites that consistently fall outside of the prediction interval would suggest aberrant behaviors and that sampling for the presence of *P*. *destructans* is warranted.

We used the model fit to *E*. *fuscus* activity data (i.e., detections classified to the species *E*. *fuscus*) to illustrate a proposed monitoring approach with a statistical model for a single WNS-susceptible species. The model was fit to data from all 41 sites to estimate among-site and among-year variation in activity, but we use the results to generate prediction intervals for four forest sites to depict how a landowner might implement this approach for WNS surveillance monitoring. Most observed activity data points fell within the 95% prediction interval, as expected (Fig 7). The prediction intervals were constructed to incorporate the estimated among-year variation in activity within a site. Future observations of activity for these sites, along with model predictions, could be plotted to compare with prediction intervals.

## Discussion

Rapidly evolving technologies to passively observe and monitor wildlife concurrent with computational improvements in processing and analyzing large quantities of data are shifting management and research techniques from an animal-in-hand approach to indirect measurements of presence, abundance, diversity, and health [45, 46]. Although acoustic monitoring has been implemented to investigate habitat relationships and community composition of bats [36], uses of this technology are expanding into other objectives. Here, we describe how year-round activity rates of bat species derived from acoustic monitoring can be used for surveillance monitoring for WNS. We developed a simple and repeatable statistical modeling approach for WNS surveillance monitoring with bat activity data collected from a broad geographic and temporal scale prior to known occurrence of WNS in the Intermountain West. This statistical approach can be applied elsewhere and may be particularly relevant for geographic locations where prominent hibernacula are inaccessible, rare, or unidentified [18].

Further, our long-term and year-round collection of acoustic data for bats expands on limited knowledge of activity rates of species through time, seasons, across locations, and prior to WNS. Very little is known about the winter ecology of many species, specifically in western North America because of the limited sampling that has occurred during that season. Technological advances in passive monitoring devices and identification software and an interest in WNS has contributed to increased monitoring for bats in winter, when previously it was assumed they were rarely active [6, 47]. Across our broad selection of 41 forested sites in Montana, we documented a diverse community of bats (13 confirmed species), including at sites with a history of forest management. Several species maintained some level of activity through winter, despite cold temperatures that averaged -1.0°C in this season, which was below temperatures where at least some insect prey could sustain flight [48]. As expected, bat activity was positively related to warmer temperatures, although the strength of the relationship varied by season, with the strongest relationships with temperatures in spring and fall. In more temperate climates, other species of bats also displayed year-round activity that varied by season, sites, and species, including both migratory and non-migratory bats [2, 6]. The implications of winter activity of bats is not well-understood, particularly as it relates to body condition and energy stores or susceptibility to WNS [6].

The ecology of bats infected with Pd may lead individuals to change behaviors, such as higher activity rates during winter when bats typically use torpor to conserve energy [6]. Aberrant behaviors by bats, including daytime activity out of roosts or hibernacula and during sub-freezing temperatures increased after detection of WNS in the southeastern U.S. [6]. However, the effects of winter bat activity on individual fitness have not been well-quantified. In North Carolina, it was posited that year-round activity of typically migratory bats, including *L*. *borealis*, whose non-migratory behaviors could increase survival by reducing mortality from threats during migration, such as strikes from wind turbines [2]. Alternatively, year-round activity by bats could be energetically costly if food resources do not meet metabolic demands and reduce reproductive output by individuals [2]. Where underground caves or human structures are uncommon as hibernacula (e.g., the Intermountain West), bats may congregate in smaller numbers in rock crevices during winter ([49]; unpublished data from this study). These wintering behaviors may provide protection against bat to bat transmission of Pd. However, talus slopes can provide suitable temperature and humidity profiles for growth of Pd, albeit below optimal levels [50]. The variability of relationships among prey availability, year-round activity, individual fitness, and susceptibility to WNS remain a knowledge gap for North American bats [51, 52].

### Surveillance monitoring for WNS

In addition to describing presence and seasonal activity rates of bats across forested sites in Montana, we developed a process for using acoustic monitoring for disease surveillance that can be applied elsewhere. Whereas approaches to incorporate uncertainty in estimates of population trends from bat count data in hibernacula are established, less information is available for managers to identify early-warnings from acoustic monitoring data [18]. Here, we suggest the same basic framework that we used for the descriptive model of bat activity could be applied to an acoustic monitoring program aimed at identifying changes in bat activity consistent with WNS. The approach could be used for single or multiple species and on individual sites or a collection of sites. Additionally, the modeling approach is useful for detecting aberrant behaviors that could be associated with WNS, including higher than normal activity rates during seasons where bats should be in torpor, or low activity rates due to population-level declines in bats. The general approach we consider is as follows:

1. Collect baseline (pre-WNS) data within a season or seasons of interest, and across several years and optionally across several sites.
2. Fit a mixed-effects model to the data, incorporating variation with temperature, among years and among sites. Optionally include other covariate information predictive of bat activity that can be easily gathered.
3. Generate a population or site-level prediction interval that includes among-year variation.
4. Monitor the collection of new data for excessive departures from the model prediction interval. In particular, look for bat activity in excess of the predicted range during winter months followed by bat activity below the predicted range during the following spring and summer [53].

We emphasize that our modeling approach was descriptive in nature and that we only recorded bat activity during nighttime. However, the approximately log-linear relationship with mean monthly temperature was strong in our dataset and may be useful in other investigations of bat activity. Bat activity also showed approximately log-linear relationships with mean daily-max and daily-min temperatures. The non-random selection of sites included in our analysis limits the interpretation of the estimated model random effects or their application to other populations of sites. Another limitation of species-specific results in this study relates to the large proportion of bat detections where no species label was assigned, where unknown biases could impact model estimates. We suggest that future site-specific surveillance models may be improved by including acoustic monitoring in daytime and by obtaining more high-quality species-specific call data with updated microphone technology [6].

We used prediction intervals to characterize expected ranges of among-year activity at the site level. One advantage of this approach in a monitoring program is that one set of intervals can be applied across multiple years without the need for annual refitting, provided suitable baseline data were used for model fitting. It is important to note that prediction intervals for mixed-effects models can be defined for any level of the random-effects structure [41], with the appropriate level dependent on the end-use application. Alternatives to prediction intervals could be employed in a monitoring program, including refitting a model each year to directly compare new data with a reference time period.

## Conclusions

Currently, most conservation and management actions related to WNS in North America are related to known hibernacula. For regions where large hibernacula are uncommon or rare, but where bats may still be susceptible to this threat, alternative approaches are necessary to ensure early detection of WNS outbreaks and prevent further losses with targeted management actions. We propose a proactive and simple monitoring approach that could assist in managing risk of further loss of bat populations, which is especially relevant given current restrictions on field research due to concerns about transmission of COVID-19 from humans to North American bats and among humans [54]. Deploying passive ARUs to quantify bat activity, developing a predictive model of activity rates for one or more sites (including managed and unmanaged forest), and examining future data for deviations from these prediction intervals can all assist with a rapid response, including WNS confirmation that could assist in recovery efforts.

## References

[1] Hammerson GA, Kling M, Harkness M, Ormes M, Young BE. Strong geographic and temporal patterns in conservation status of North American bats. Biol Conserv. 2017;212:144–52.

[2] Grider JF, Larsen AL, Homyack JA, Kalcounis-Rueppell MC. Winter activity of coastal plain populations of bat species affected by white-nose syndrome and wind energy facilities. PLoS One. 2016;11(11):e0166512 10.1371/journal.pone.0166512 27851832 PMC5112809

[3] Department of Interior FaWS. White-nose syndrome: The devastating disease of hibernating bats in North America. Washington, D.C.; 2014.

[4] Cryan PM, Meteryer CU, Boyles JG, Blehert DS. Wing pathology of white-nose syndrome in bats suggests life-threatening disruption of physiology. BMC Biol. 2010;125:1–8. 10.1186/1741-7007-8-135 PMC2984388 21070683

[5] Reeder DM, Frank CL, Turner GG, Meteryer CU, Kurta A, Britzke ER, et al Frequent arousal from hibernation linked to severity of infection and mortality in bats with white-nose syndrome. PLoS One. 2012;7:e38920 10.1371/journal.pone.0038920 22745688 PMC3380050

[6] Bernard RF, McCracken GF. Winter behavior of bats and the progression of white-nose syndrome in the southeastern United States. Ecol Evol. 2017;7(5):1487–96. 10.1002/ece3.2772 28261459 PMC5330875

[7] Department of the Interior, Fish and Wildlife Service,. Endangered and Threatened Wildlife and Plants; 4(d) Rule for the Northern Long-Eared Bat. Federal Register. 2016;81(9):1900–22.

[8] Government of Canada. Bats and white-nose syndrome 2020 [Available from: https://www.canada.ca/en/environment-climate-change/services/species-risk-education-centre/fact-sheets/bats-white-nose-syndrome.html.

[9] Verant ML, Bohuski EA, Richgels KLD, Olival KJ, Epstein JH, Blehert DS. Determinants of *Pseudogymnoascus destructans* within bat hibernacula: Implications for surveillance and management of white- nose syndrome. J Appl Ecol. 2017;55:820–9. 10.1111/1365-2664.13070 PMC5877478 29610540

[10] Maher SP, Kramer AM, Pulliam JT, Zokan MA, Bowden SE, Barton HD, et al Spread of white-nose syndrome on a network regulated by geography and climate. Nat Commun. 2012;3:1306 10.1038/ncomms2301 23250436

[11] Montana Fish W, & Parks. Fungus that causes white-nose syndrome found in eastern Montana 2020 [Available from: http://fwp.mt.gov/news/newsReleases/fishAndWildlife/nr_1369.html.

[12] Washington Department of Fish and Wildlife. Bat-killing disease white-nose syndrome confirmed east of the Cascade Range in Washington 2019 [Available from: https://wdfw.wa.gov/news/bat-killing-disease-white-nose-syndrome-confirmed-east-cascade-range-washington.

[13] Lorch JM, Palmer JM, Lindner DL, Ballmann AE, George KG, Griffin K, et al First detection of bat white-nose syndrome in Western North America. mSphere. 2016;1(4). 10.1128/mSphere.00148-16 PMC4973635 27504499

[14] Boyles JG, Dunbar MB, Whitaker JO Jr. Activity following arousal in winter in North American vespertilionid bats. Mamm Rev. 2006;36:267–80.

[15] Bogan MA, Cryan PM, Valdez EW, Ellison LE, O'Shea TJ. Western crevice and cavity roosting bats. Fort Collins, Colorado: U.S. Geological Survey; 2003.

[16] Falxa G. Winter foraging of silver-haired and California myotis bats in western Washington. Northwest Nat. 2007;88:98–100.

[17] Weller TJ, Rodhouse TJ, Neubaum DJ, Ormsbee PC, Dixon RD, Popp DL, et al A review of bat hibernacula across the western United States: Implications for white-nose syndrome surveillance and management. PLoS One. 2018;13(10):e0205647 10.1371/journal.pone.0205647 30379854 PMC6209190

[18] Ingersoll TE, Sewall BJ, Amelon SK. Improved analysis of long-term monitoring data demonstrates marked regional declines of bat populations in the eastern United States. PLoS One. 2013;8(6):e65907 10.1371/journal.pone.0065907 23805192 PMC3689752

[19] Bender MJ, Castleberry SB, Miller DA, Bently Wigley T. Site occupancy of foraging bats on landscapes of managed pine forest. For Ecol Manage. 2015;336:1–10.

[20] Vindigni MA, Morris AD, Miller DA, Kalcounis-Ruepell MC. Use of modified water sources by bats in a managed pine landscape. For Ecol Manage. 2009;258:2056–61.

[21] Brigham RM. Bats in forests: what we know and what we need to learn In: Lacki MJ, Hayes JP, Kurta A, editors. Bats in Forests: Conservation and Management. Baltimore, Maryland: Johns Hopkins University Press; 2007.

[22] Owen SF, Menzel MA, Edwards JW. Bat activity in harvested and intact forest stands in the Allegheny Mountains. Northern Journal of Applied Forestry. 2004;21(3):154–9.

[23] Jenkins CN, Van Houtan KS, Pimm SL, Sexton JO. US protected lands mismatch biodiversity priorities. Proceedings of the National Academy of Sciences of the United States of America. 2015;112(16):5081–6. 10.1073/pnas.1418034112 25847995 PMC4413281

[24] Rodhouse TJ, Philippi TE, Monahan W, B., Castle KT. A macroecological perspective on strategic bat conservation in the U.S. National Park Service. Ecophere. 2016;7(11):1–21.

[25] Cox MR, Willcox EV, Keyser PD, Vander Yacht AL. Bat response to prescribed fire and overstory thinning in hardwood forest on the Cumberland Plateau, Tennessee. For Ecol Manage. 2016;359:221–31.

[26] Burns LKL, Loeb SC, Bridges JWC. Effects of fire and its severity on occupancy of bats in mixed pine-oak forests. For Ecol Manage. 2019;446:151–63.

[27] Pfister RD, Kovalchik BL, Arno SF, Presby RC. Forest habitat types of Montana. Ogden, Utah, USA: U.S. Department of Agriculture, Forest Service, Intermountain Forest and Range Experiment Station; 1977.

[28] Omernik JA. Ecoregions of the conterminous United States. Annals of the American Association of Geographers. 1987;77:118–25.

[29] Arno SF. Forest regions of Montana Ogden, UT; 1979.

[30] Montana land cover/Land use theme [Internet]. Montana Natural Heritage Program. 2017 [cited 28 May 2020]. Available from: ftp://ftp.geoinfo.msl.mt.gov/Data/Spatial/MSDI/LandUse_LandCover/Montana_LandCover.zip.

[31] Maxell BA. Montana bat and white‐nose syndrome surveillance plan and protocols 2012 ‐2016. Helena, Montana, USA; 2015.

[32] Bachen D. A directory of reports on long-term acoustic monitoring for bats at sites across the Northern Rocky Mountains and Great Plains. Helena, Montana, USA: Montana Natural Heritage Program; 2020.

[33] Adams AM, Jantzen MK, Hamilton RM, Fenton MB. Do you hear what I hear? Implications of detector selection for acoustic monitoring of bats. Methods in Ecology and Evolution. 2012;3:992–8.

[34] Goerlitz HR. Weather conditions determine attenuation and speed of sound: Environmental limitations for monitoring and analyzing bat echolocation. Ecology and Evolution. 2018;8:5090–100. 10.1002/ece3.4088 29876084 PMC5980448

[35] Loeb SC, Rodhouse TJ, Ellison LE, Lausen CL, Reichard JD, Irvine KM, et al. A plan for the North American bat monitoring program (NABat). Asheville, North Carolina, USA: Southern Research Station; 2015. Contract No.: General Technical Report SRS-208.

[36] Gannon WL, Sherwin RE, Haymound S. On the importance of articulating assumptions when conducting acoustic studies of habitat use by bats. Wildl Soc Bull. 2003;31:45–61.

[37] Maxell BA. Montana Bat and White-Nose Sydrome Surveillance Plan and Protocols 2012–2016. Helena, Montana USA: Montana Natural Heritage Program; 2015.

[38] R Core Team. R: A language and environment for statistical computing. R Foundation for statistical Computing Vienna, Austria 2019.

[39] Bates PJ, DebRoy S, Sarkar D, Team RC. nlme: Linear and Nonlinear Mixed Effects Models. R package version 3.1–139 ed2019.

[40] Hahn GJ, Meeker WQ. Statistial intervals: A guide for practitioners New York: John Wiley and Sons; 2011.

[41] Knowles JE, Frederick C. merTools: Tools for Analyzing Mixed Effect Regression Models. R package version 0.5.0 ed2019.

[42] White-nose Syndrome Response Team. Bats affected by WNS 2020 [Available from: https://www.whitenosesyndrome.org/static-page/bats-affected-by-wns.

[43] Frank C, Michalski A, McDonough A, Rahimian M, Rudd R, Herzog C. The resistance of a North American bat species (*Eptesicus fuscus*) to white-nose syndrome (WNS). PLOS One. 2014;9:e113958 10.1371/journal.pone.0113958 25437448 PMC4250063

[44] Frick WF, J.F. P, A.C. H, Langwig KE, Reynolds DS, Turner GG, et al An emerging disease causes regional population collapse of a common North American bat species. Science. 2010;329:679–82. 10.1126/science.1188594 20689016

[45] Gomes C, Dietterich T, Barrett C, Conrad J, Dilkina B, Ermon S, et al Computational sustainability: computing for a better world anda sustainable future. Communications of the Association for Computing Machinery. 2019;62:56–65.

[46] Darling JA. How to learn to stop worrying and love eDNA monitoring. Aquat Ecosyst Health Manage. 2019(2019):1–13. 10.1080/14634988.2019.1682912 PMC7751714 33364913

[47] Schwab NA, Mabee TJ. Winter acoustic activity of bats in Montana. Northwest Nat. 2014;95:13–27.

[48] Reinhold JM, Lazzari CR, Lahondere C. Effects of the environmental temperature on *Aedes aegypti* and *Aedes albopictus* mosquitoes: a review. Insects. 2018;2018(9):1–17. 10.3390/insects9040158 PMC6316560 30404142

[49] Lausen CL, Barclay RMR. Winter bat activity in the Canadian prairies. Canadian Journal of Zoology. 2006;84:1079–86.

[50] Neubaum DJ. Unsuspected retreats: autumn transitional roosts and presumed winter hibernacula of little brown myotis in Colorado. J Mammal. 2018;99:1294–306.

[51] Johnson JS, Lacki MJ, Thomas SC, Grider JF. Frequent arousals from winter torpor in Rafinesque’s big-eared bat (*Corynorhinus rafinesquii*). PLoS One. 2012;7:1–11. 10.1371/journal.pone.0049754 PMC3504087 23185427

[52] Bernard R, Reichard JD, Coleman JTH, Blackwood JC, Verant ML, Segers JL, et al Identifying research needs to inform white-nose syndrome management decisions. Conservation Science and Practice. 2020;e2020:1–17.

[53] Dzal Y, McGuire LP, Veselka N, Fenton MB. Going, going, gone: the impact of white-nose syndrome on the summer activity of the little brown bat (*Myotis lucifugus*). Biol Lett. 2011;7:392–4. 10.1098/rsbl.2010.0859 21106570 PMC3097845

[54] Fish & Wildlife Health Committee and Wildlife Resource Policy Committee, Bat Working Group, Guidance for bat-related activities in response to Covid-19. Association of Fish and Wildlife Agencies; 2020.

